# Supplementary material for: Dissociation between individual differences in self-reported pain intensity and underlying fMRI brain activation
Source: Nat Commun. 2022 Jun 22;13:3569. doi: 10.1038/s41467-022-31039-3 (PMC9218124; doi:10.1038/s41467-022-31039-3)
Supplement: Supplementary file 1 — Supplementary Information [file 41467_2022_31039_MOESM1_ESM.pdf]

Supplementary figure 1. Stimulus-response data revealed three classes of pain sensitivity.

Participants from the Low (A) and Moderate (B) Pain Sensitivity classes had large exponents and low proportionality constants (Low: intensity: constant = -9.79, exponent = 3.88; unpleasantness: constant = -12.1, exponent = 4.74; Moderate: intensity: constant = -10.02, exponent = 4.46; unpleasantness: constant = -11.84, exponent = 5.12) , while participants from the High Pain Sensitivity Class (C) had greater constants than those of the two other classes (intensity: constant = -5.49; unpleasantness: constant = -6.88) and the smallest exponent (intensity: exponent = 2.93; unpleasantness: exponent = 3.45). Error bars represent standard error of the mean. ˚C represent degree Celsius. Source data are provided as an xlsx Source Data file.

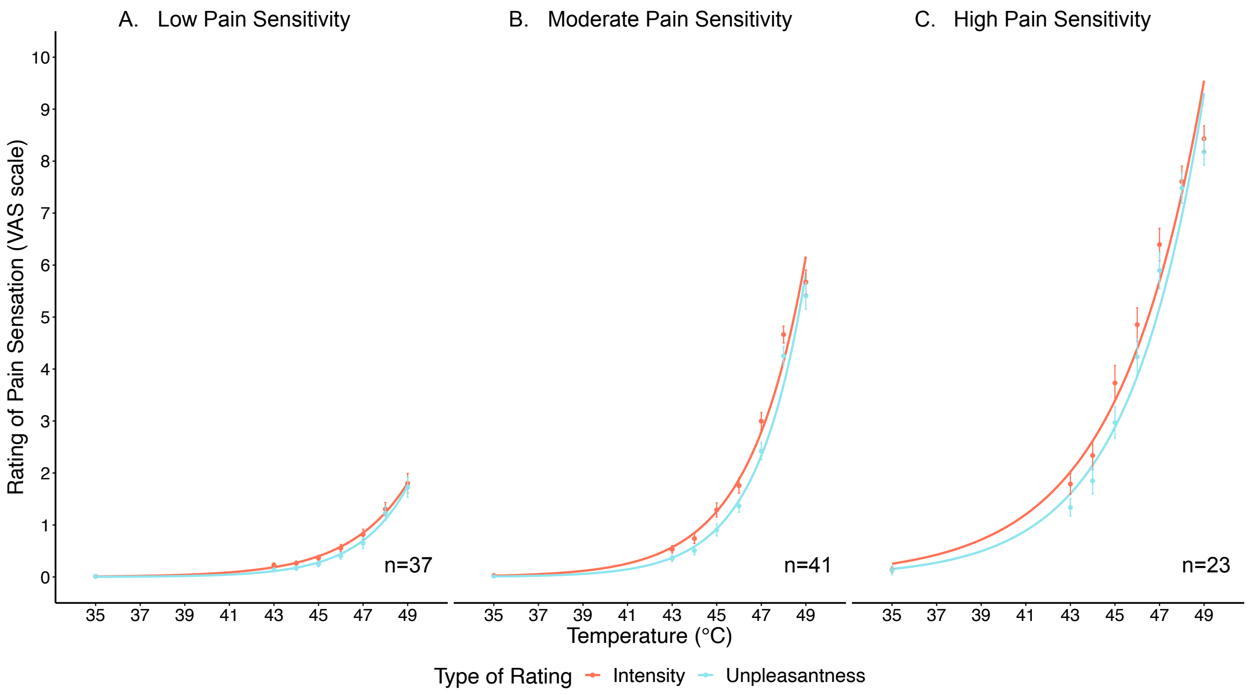

Supplementary figure 2. Individual ratings of pain intensity in response to high intensity heat stimulation sorted in ascending order. Individual averages of pain intensity ratings in response to 48˚C stimulation are presented in ascending order with error bars indicating individual standard error of the mean. n = 101 participants. Source data are provided as an xlsx Source Data file.

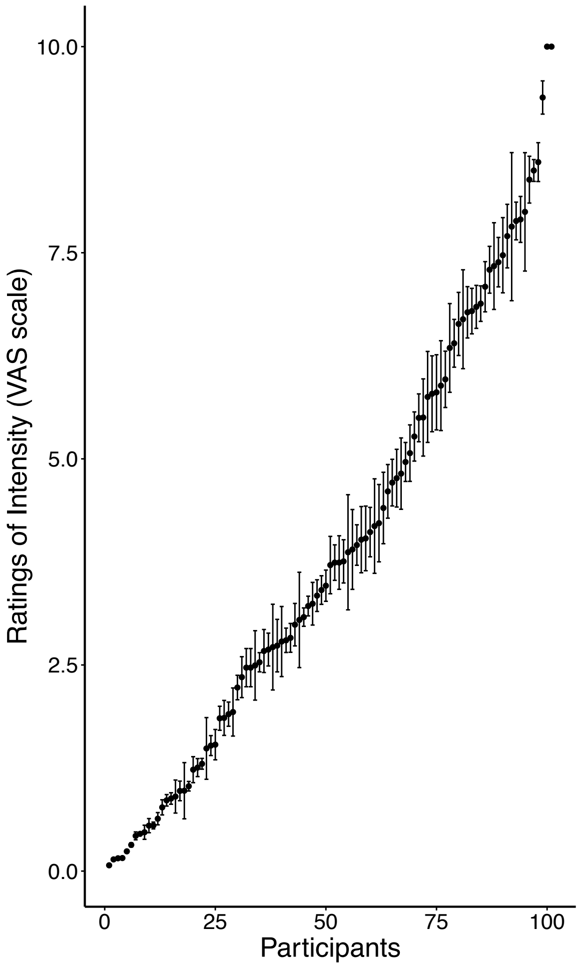

Supplementary figure 3. Positive individual NPS expression is not correlated to individual ratings of perceived pain intensity in response to high intensity heat stimulus (48°C). A) All participants showed positive individual NPS expression, with a mean of 2723.89 (bar) and standard error of 103.47 (error bar). B) There was no relationship between the level of NPS expression and the perceived pain intensity, supporting the results of the univariate analysis. Source data are provided as an xlsx Source Data file.

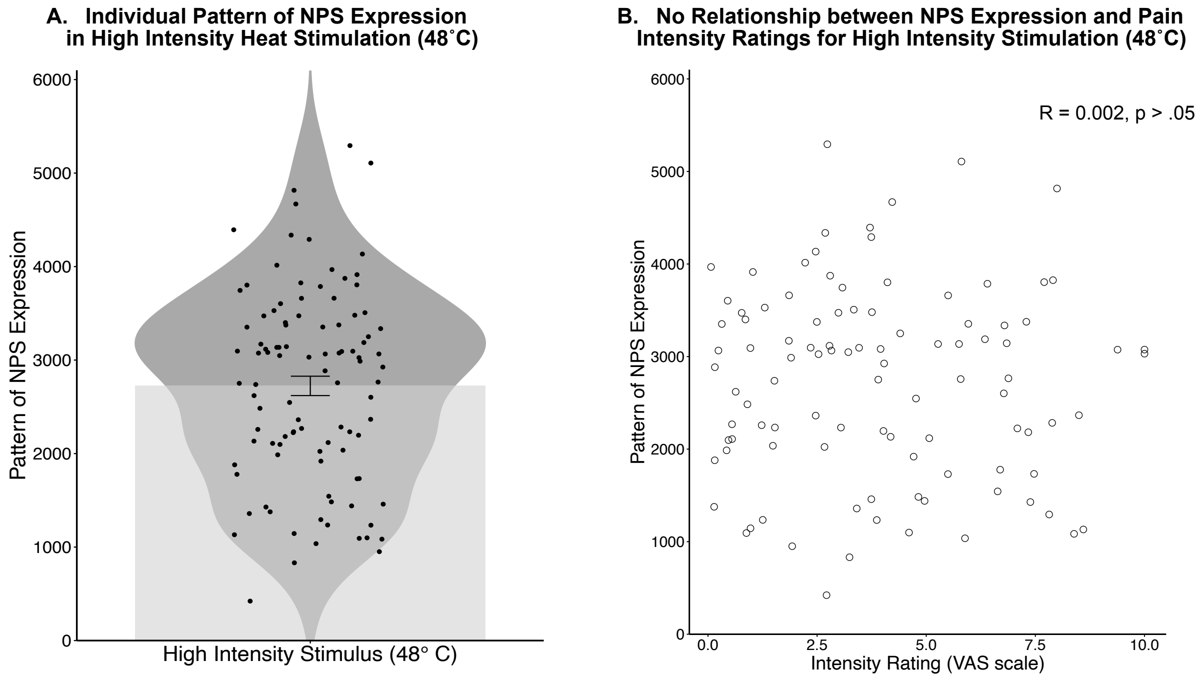

Supplementary Figure 4. No relationship between perceived pain intensity and brain activation using multivariate LASSO-PCR. A multivariate regression model using LASSO-PCR was trained on our data to determine if perceived pain intensity was related to brain activation associated with high intensity heat stimuli. Results show that the model failed to show a relationship between perceived pain intensity and brain activation. Source data are provided as an xlsx Source Data file.

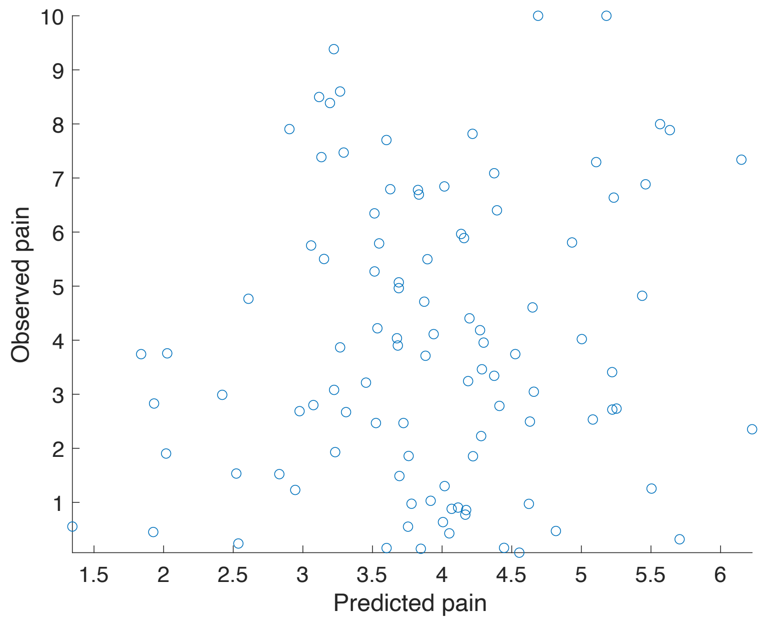

Supplementary figure 5. Effect of the graded increase in intensity of cold stimulation on brain activation. A) Average ratings of pain intensity associated with cold stimulation. B, C, and D). Increased brain activation in response to high (0.5°C, B) and low (3°C, C) intensity cold stimulation and differences between the two intensities of stimulation (D) are observed in areas such as the putamen (Put), caudate nucleus (Cau), the primary somatosensory cortex (SI), the secondary somatosensory cortex (SII), the insula (Ins), the anterior cingulate cortex (ACC), and dorsolateral prefrontal cortex (DLPFC). Decreased activation in response to the same stimuli is especially present in the precuneus (prec) and the posterior cingulate cortex (PCC). n = 73 participants. Source data are provided as an xlsx Source Data file.

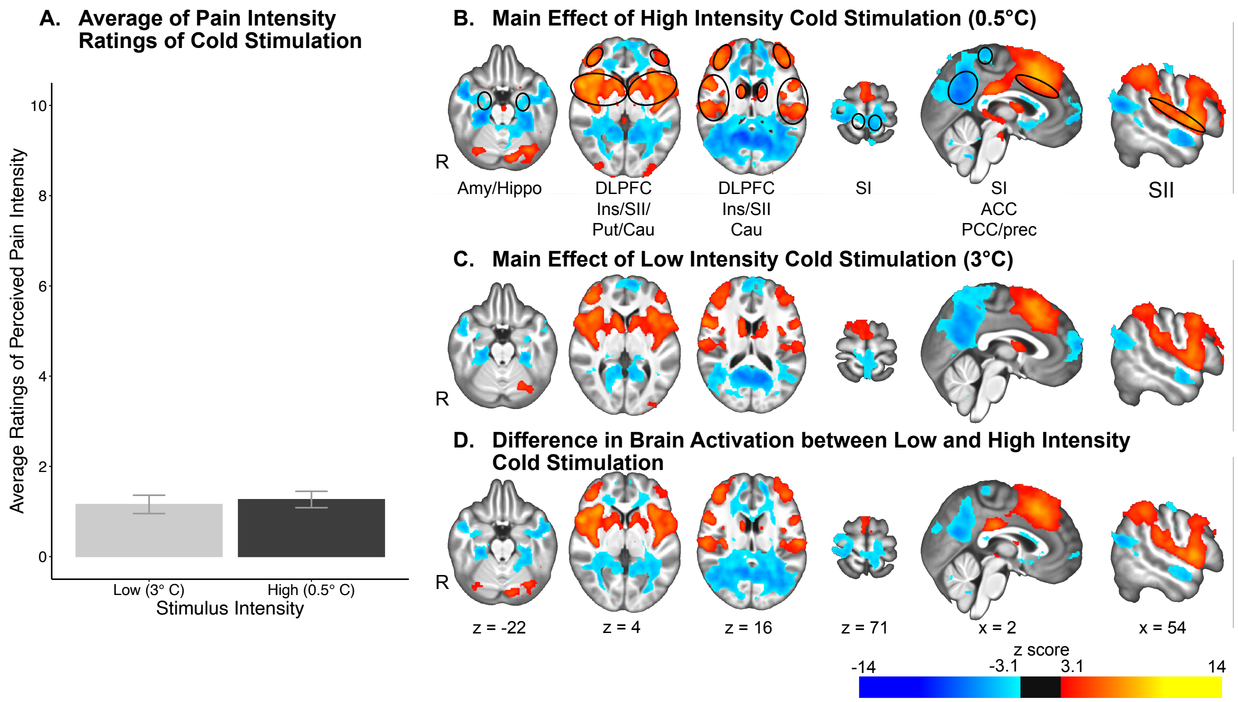

Supplementary figure 6. Effect of the graded increase in intensity of auditory stimulation on brain activation. A) Average ratings of intensity associated with auditory stimulation. B, C, and D) Increased brain activation in response to high (90dB, B) and low (80dB, C) intensity auditory stimuli and differences between the two intensities of stimuli (D) are observed in areas such as the putamen (Put), caudate nucleus (Cau), the primary somatosensory cortex (SI), the secondary somatosensory cortex (SII), the primary auditory cortex (AI), the insula (Ins), the anterior cingulate cortex (ACC), and dorsolateral prefrontal cortex (DLPFC). Decreased activation in response to the same stimuli is especially present in the amygdala and hippocampus (Amy/Hippo), in the precuneus (prec) and the posterior cingulate cortex (PCC). n = 97 participants. Source data are provided as an xlsx Source Data file.

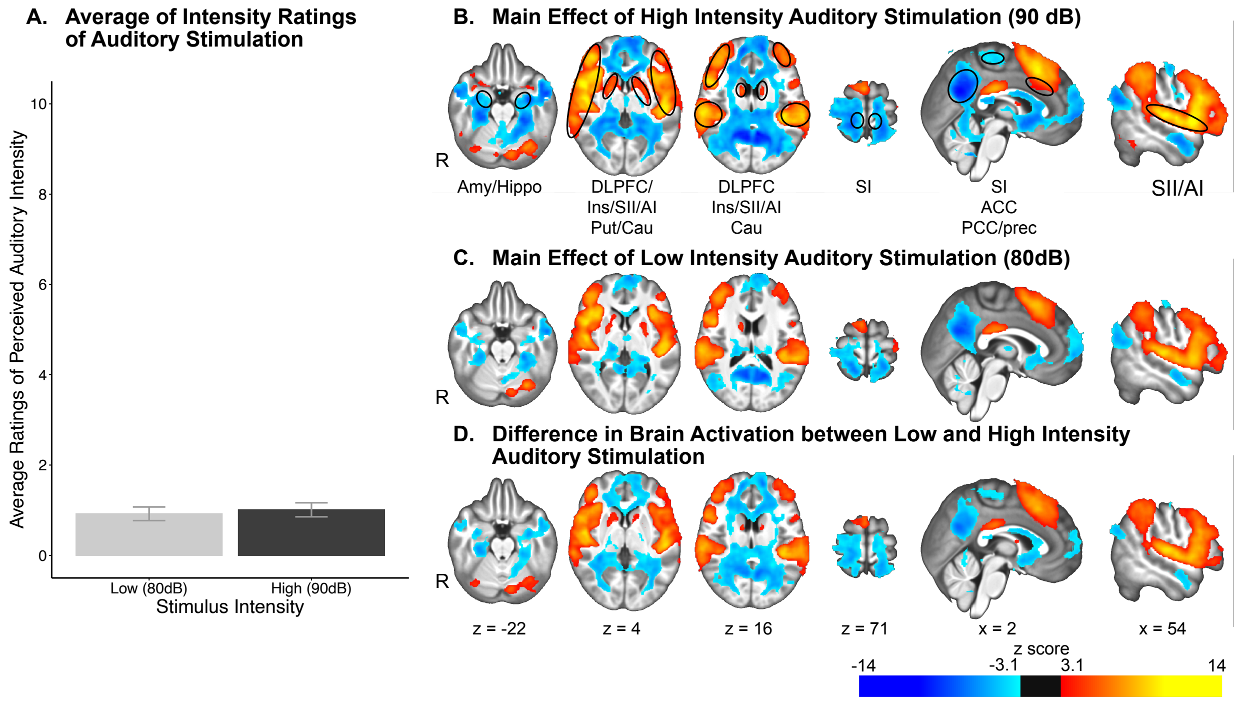

Supplementary table 1. No difference between classes of pain sensitivity in demographics and psychological factors. Source data are provided as an xlsx Source Data file.

	Low pain sensitivity (N=37)	Moderate pain sensitivity (N=41)	High pain sensitivity (N=23)	Overall (N=101)
**Sex**				
female	22 (59.5%)	24 (58.5%)	12 (52.2%)	58 (57.4%)
male	15 (40.5%)	17 (41.5%)	11 (47.8%)	43 (42.6%)
**Age (years)**				
Mean (SD)	28.3 (7.16)	27.5 (7.80)	30.5 (8.39)	28.5 (7.72)
Median [Min, Max]	28.0 [16.0, 43.0]	27.0 [15.0, 44.0]	33.0 [15.0, 44.0]	28.0 [15.0, 44.0]
**Score in mindfulness**				
Mean (SD)	41.5 (6.08)	40.7 (6.62)	43.1 (6.49)	41.5 (6.36)
Median [Min, Max]	42.0 [27.0, 52.0]	41.5 [23.0, 52.0]	42.0 [31.0, 56.0]	42.0 [23.0, 56.0]
**Score in anxiety**				
Mean (SD)	11.4 (5.27)	11.5 (5.51)	12.2 (3.65)	11.6 (5.03)
Median [Min, Max]	10.5 [0, 21.0]	10.0 [0, 25.0]	12.0 [8.00, 19.0]	10.0 [0, 25.0]
**Score in depression**				
Mean (SD)	10.2 (4.14)	9.37 (3.58)	9.07 (2.02)	9.62 (3.55)
Median [Min, Max]	9.00 [0, 21.0]	9.00 [0, 19.0]	9.00 [8.00, 16.0]	9.00 [0, 21.0]
**Score in catastrophizing**				
Mean (SD)	5.89 (7.38)	7.83 (8.84)	6.13 (8.00)	6.74 (8.07)
Median [Min, Max]	3.00 [0, 25.0]	5.00 [0, 39.0]	2.00 [0, 28.0]	4.00 [0, 39.0]
**Score in sleepiness**				
Mean (SD)	4.76 (3.17)	5.51 (3.59)	6.43 (3.24)	5.45 (3.39)
Median [Min, Max]	4.00 [0, 13.0]	5.00 [1.00, 14.0]	7.00 [1.00, 15.0]	5.00 [0, 15.0]
**Score in impulsiveness**				
Mean (SD)	51.3 (8.34)	54.8 (9.19)	55.0 (7.47)	53.5 (8.61)
Median [Min, Max]	53.0 [35.0, 68.0]	54.0 [38.0, 77.0]	53.0 [41.0, 71.0]	53.0 [35.0, 77.0]
**Score in sleep quality**				
Mean (SD)	3.96 (2.52)	4.80 (2.20)	4.33 (2.23)	4.38 (2.33)
Median [Min, Max]	3.50 [1.00, 11.0]	5.00 [1.00, 9.00]	5.00 [0, 8.00]	4.00 [0, 11.0]
**Score in positive affect**				
Mean (SD)	35.0 (7.61)	33.1 (7.94)	31.0 (8.03)	33.3 (7.90)
Median [Min, Max]	35.0 [20.0, 47.0]	34.0 [18.0, 50.0]	32.0 [14.0, 44.0]	34.0 [14.0, 50.0]
**Score in negative affect**				
Mean (SD)	13.0 (4.25)	15.7 (7.04)	15.0 (4.53)	14.5 (5.68)
Median [Min, Max]	11.0 [10.0, 25.0]	14.0 [10.0, 50.0]	14.0 [10.0, 29.0]	13.0 [10.0, 50.0]
**Score in pain interference**				
Mean (SD)	8.25 (0.585)	9.23 (2.53)	8.93 (1.22)	8.79 (1.79)
Median [Min, Max]	8.00 [8.00, 10.0]	8.00 [8.00, 18.0]	8.00 [8.00, 11.0]	8.00 [8.00, 18.0]

Supplementary table 2. Peak positive brain activation associated with high intensity heat stimuli. Activation location was defined using the Harvard-Oxford Cortical and Subcortical Structural Atlas (H) and the Juelich Histological Atlas (J). R: right; L: left

Cluster Index	Z	x	y	z	Atlas Location (H/J)
6	13.5	54	6	6	Precentral Gyrus / Broca's area BA44 R
6	13	56	0	8	Central Opercular Cortex / Secondary somatosensory cortex
6	12.8	34	4	10	Insular Cortex
6	12.8	40	0	8	Insular Cortex / Secondary somatosensory cortex
6	12.7	40	10	6	Central Opercular Cortex
6	12.7	40	2	4	Insular Cortex
5	9.47	-32	46	30	Frontal Pole
4	8.61	0	-26	28	Posterior Cingulate Gyrus
3	5.39	56	-62	4	Lateral Occipital Cortex, inferior/ Visual cortex V5 R
2	5.42	-8	-80	42	Cuneal Cortex / Superior parietal lobule 7P L
2	4	-8	-80	52	Lateral Occipital Cortex, superior division / Superior parietal lobule 7P L
1	5.27	12	-76	42	Precuneous Cortex / Superior parietal lobule 7P R
1	3.33	16	-72	52	Superior Lateral Occipital Cortex / Superior parietal lobule 7P R

Supplementary table 3. Peak negative brain activation associated with high intensity heat stimuli

Cluster Index	Z	x	y	z	Atlas Location (H/J)
3	12.6	10	-54	18	Precuneous Cortex
3	12.6	6	-58	22	Precuneous Cortex
3	12.5	30	-32	-20	Posterior Temporal Fusiform Cortex
3	12.4	-6	-60	20	Precuneous Cortex
3	12.3	-10	-54	16	Precuneous Cortex / Cingulum L
3	12.3	-40	-72	36	Superior Lateral Occipital Cortex / Inferior parietal lobule PGp L
2	6.09	-44	-30	70	2.0% Postcentral Gyrus
2	4.52	-54	-16	58	18% Postcentral Gyrus
2	3.21	-44	-24	46	Postcentral Gyrus / Primary somatosensory cortex BA3b L
1	5.64	-68	-4	28	Postcentral Gyrus, Precentral Gyrus / Primary somatosensory
1	5.37	-64	-6	34	Postcentral Gyrus
1	5.36	-66	-2	32	Precentral Gyrus
1	5.17	-62	-6	28	Postcentral Gyrus / Primary somatosensory cortex BA3b L,BA1 L

Supplementary table 4. Peak positive brain activation associated with high intensity cold stimuli

Cluster Index	Z	x	y	z	Atlas Location (H/J)
8	8.43	40	-2	-6	Insular cortex / Inferior occipito-frontal fascicle R
8	8.4	56	10	4	Inferior Frontal Gyrus, pars opercularis / Broca's area BA44 R
8	8.27	-42	14	-2	Insular cortex
8	8.14	40	18	0	Insular cortex
8	8.07	-42	0	-2	Insular cortex / Acoustic radiation L
8	8.03	-60	-20	20	Postcentral Gyrus / Secondary somatosensory cortex, Parietal operculum OP1 L
7	8.51	0	18	44	Paracingulate Gyrus
7	8.45	-2	22	42	Paracingulate Gyrus
7	6.65	2	-24	30	Posterior Cingulate Gyrus
7	5.41	6	-2	72	Juxtapositional Lobule Cortex / Premotor cortex BA6 R
7	5.4	4	4	72	Juxtapositional Lobule Cortex / Premotor cortex BA6 R
7	4.83	-6	-6	70	Juxtapositional Lobule / Premotor cortex BA6 R
6	7.03	-38	-58	-30	Cerebellum
6	6.66	-32	-72	-22	Occipital Fusiform Gyrus, Cerebellum
6	6.02	-10	-82	-22	Occipital Fusiform Gyrus
6	6.01	-10	-80	-26	Occipital Fusiform Gyrus
6	5.62	-38	-76	-26	Lateral Occipital Cortex, inferior division
6	5.43	-48	-60	-34	Cerebellum
5	5.5	34	-70	-24	Occipital Fusiform Gyrus
5	4.39	38	-60	-28	Cerebellum
5	4.29	40	-56	-32	Cerebellum
5	4.15	44	-58	-30	Cerebellum
5	4.1	48	-58	-30	Cerebellum
5	3.96	50	-64	-32	Cerebellum
4	4.78	-40	-24	52	Postcentral Gyrus / Primary somatosensory cortex BA3b L
4	4.07	-40	-20	60	Precentral Gyrus / Premotor cortex BA6 L
3	4.27	14	-98	-6	Occipital Pole / Visual cortex V1 BA17 R
3	4.06	14	-102	-2	Occipital Pole / Visual cortex V1 BA17 R
3	4.02	20	-98	-8	Occipital Pole / Visual cortex V1 BA17 R
3	3.97	16	-94	-10	Occipital Pole / Visual cortex V1 BA17 R
3	3.96	26	-92	-14	Occipital Pole / Visual cortex V3V R
3	3.85	24	-98	-8	Occipital Pole / Visual cortex V1 BA17 R
2	4.84	36	-96	4	Occipital Pole / Visual cortex V3V R
2	4.28	38	-90	0	Inferior Lateral Occipital Cortex / Visual cortex V4 R
2	4.15	36	-92	6	Occipital Pole / Visual cortex V3V R
2	3.82	40	-92	-6	Occipital Pole / Visual cortex V3V R
2	3.51	36	-98	-4	Occipital Pole / Visual cortex V2 BA18 R
1	4.68	22	32	-14	Frontal Orbital Cortex
1	4.42	30	40	-12	Frontal Pole
1	4.35	24	42	-14	Frontal Pole

Supplementary table 5. Peak negative brain activation associated with high intensity cold stimuli

Cluster Index	Z	x	y	z	Atlas Location (H/J)
1	8.76	12	-52	20	Precuneous Cortex / Cingulum R
1	8.46	22	-54	20	Precuneous Cortex / Callosal body
1	8.41	8	-58	18	Precuneous Cortex / Visual cortex V2 BA18 R
1	8.35	34	-42	-10	Temporal Occipital Fusiform Cortex / Optic radiation R
1	8.33	12	-50	12	Precuneous Cortex / Cingulum R
1	8.21	24	-36	-12	Parahippocampal Gyrus, posterior division / Hippocampus subiculum R

Supplementary table 6. Peak positive brain activation associated with high intensity auditory stimuli

Cluster Index	Z	x	y	z	Atlas Location (H/J)
4	11.6	-48	-26	10	Heschel's Gyrus L / Primary Auditory TE1.0L
4	10.8	-40	-30	12	Planum Temporale/ Primary Auditory TE1.0L
4	10.6	52	8	2	Central Opercular Cortex or Precentral Gyrus / Brocas BA44
4	10.4	50	-22	8	Heschel's Gyrus R / Primary Auditory TE1.0R
4	10.4	44	-6	-6	Insular Cortex / Insula Id1R
4	10.3	48	-6	-2	Heschel's Gyrus, Planum Polare / Primary Auditory TE1.2R
3	8.29	-32	-62	-30	R amygdala
3	8.03	-30	-70	-24	Cerebellum
3	6.81	-10	-76	-30	Cerebellum
3	6.53	-10	-78	-22	Occipital Fusiform Gyrus
3	6.08	-46	-60	-32	Cerebellum
3	4.22	-40	-82	-20	Inferior Lateral Occipital Cortex / Visual Cortex V4L
2	8.31	-2	-26	28	Posterior Cingulate Gyrus
1	5.22	12	-76	42	Precuneous Cortex / Superior parietal lobule 7PR
1	4.5	10	-80	52	Superior Lateral Occipital Cortex / Superior Parietal Lobule 7PR

Supplementary table 7. Peak negative brain activation associated with high intensity auditory stimuli

Cluster Index	Z	x	y	z	Atlas Location (H/J)
1	10.9	8	-56	20	Precuneous Cortex
1	10.9	4	-64	24	Precuneous Cortex / Superior parietal lobule 7MR
1	10.6	-6	-62	20	Precuneous cortex / Superior parietal lobule 7ML

1	10.4	-14	-60	22	Precuneous Cortex / Visual Cortex V2 BA18 L
1	10.3	16	-58	22	Precuneous Cortex
1	10.3	-6	-58	18	Precuneous Cortex

Supplementary table 8. Peak positive brain activation associated with intensity and high intensity auditory stimuli

Cluster Index	Z	x	y	z	Atlas Location (H/J)
6	4.87	-54	0	2	Central Opercular Cortex / Secondary Somatosensory Cortex, Parietal operculum OP4L
6	4.83	-48	6	2	Central opercular cortex / Brocas BA44
6	4.59	-44	16	4	Inferior frontal gyrus, pars opercularis / Brocas BA44
6	4.36	-32	18	4	Insular cortex
6	4.24	-40	22	-2	Frontal operculum / Brocas BA45
6	4.19	-50	12	-2	Inferior frontal gyrus, pars opercularis / Brocas BA45
5	5.2	-68	-14	10	Postcentral gyrus
5	4.9	-70	-10	6	Superior temporal gyrus
5	4.72	-56	-34	18	Planum temporale, Parietal operculum cortex / Inferior parietal lobule
5	4.55	-46	-34	18	Parietal operculum cortex / Inferior parietal lobule
5	4.52	-56	-26	16	Parietal operculum cortex / Secondary somatosensory cortex, parietal operculum OP1L
5	4.34	-64	-22	14	Central opercular cortex / Secondary somatosensory cortex, parietal operculum OP1L
4	4.42	56	10	-2	Temporal pole / Brocas BA44 R
4	4.24	56	-2	14	Central opercular cortex / Secondary somatosensory cortex, parietal operculum OP4R
4	4.18	60	-6	6	Central opercular cortex / Primary auditory cortex
4	4.11	56	-4	8	Central opercular cortex / secondary somatosensory cortex, parietal operculum OP4R
4	3.78	64	-12	10	Central opercular cortex / secondary somatosensory cortex, parietal operculum OP4R
4	3.7	60	4	6	Precentral gyrus / Primary auditory cortex
3	4.84	56	-28	14	Planum temporale / Inferior parietal lobe
3	4.27	46	-32	16	Planum temporale / Secondary somatosensory cortex, parietal operculum op1R
3	3.86	48	-28	10	Planum temporale / Primary auditory cortex
2	5.43	40	-58	-28	Cerebellum
2	4.19	32	-54	-32	Cerebellum
1	4.38	-36	-22	0	Insular cortex / Insula
1	4.31	-40	-10	-12	Planum polare / Insula
1	4.2	-44	-18	-2	Planum polare / Insula
1	3.48	-38	-4	-14	Insular cortex / Inferior occipito-frontal fascicle L

Supplementary table 9. Peak negative brain activation associated with intensity and high intensity auditory stimuli

Cluster Index	Z	x	y	z	Atlas Location (H/J)
6	5.19	0	-58	26	Precuneous cortex / Superior parietal lobule
6	4.91	-12	-48	26	Posterior cingulate gyrus
6	4.89	10	-58	28	Precuneous cortex
6	4.73	-12	-58	34	Precuneous cortex
6	4.57	-6	-52	28	Posterior cingulate gyrus
6	4.5	0	-40	20	Posterior cingulate gyrus
5	4.93	8	50	34	Superior frontal gyrus
5	4.43	-2	62	22	Frontal pole
5	4.34	-18	44	36	Frontal pole
5	4.33	18	44	30	Frontal pole
5	4.29	8	60	34	Frontal pole
5	4.24	6	56	24	Superior frontal gyrus
4	4.75	-28	-24	-14	Hippocampus dentate gyrus L
4	4.53	-46	-14	-18	Anterior middle temporal gyrus
4	4.43	-28	-48	-16	Temporal occipital fusiform cortex
4	4.34	-28	-44	-18	Temporal occipital fusiform cortex
4	4.24	-20	-36	-18	Posterior parahippocampal gyrus
4	4.01	-26	-14	-16	Hippocampus cornu ammonis L
3	4.47	-42	-72	40	Superior lateral occipital cortex / Inferior parietal lobule PGpL
3	4.28	-36	-66	36	Superior lateral occipital cortex / Inferior parietal lobule PgaL
2	4.56	-28	26	38	Middle Frontal Gyrus
2	3.97	-32	16	44	Middle Frontal Gyrus
2	3.95	-24	34	40	Middle Frontal Gyrus
2	3.57	-20	28	34	Superior Frontal Gyrus
2	3.48	-22	30	26	Superior Frontal Gyrus
2	3.32	-40	18	48	Middle Frontal Gyrus / Broca's area BA44 L
1	4.06	14	46	6	Paracingulate Gyrus
1	3.81	10	34	14	Anterior cingulate gyrus / Cingulum R
1	3.74	10	46	2	Paracingulate gyrus
1	3.71	12	42	10	Anterior cingulate gyrus
1	3.65	8	46	6	Paracingulate Gyrus

Supplementary table 10. Peak positive brain activation associated with low intensity heat stimuli

Cluster Index	Z	x	y	z	Atlas Location (H/J)
4	13.7	-56	-22	16	Central Opercular Cortex / Secondary somatosensory cortex, Parietal operculum OP1 L
4	12.6	6	16	42	Paracingulate Gyrus / Premotor cortex BA6 R
4	12.4	54	6	6	Precentral Gyrus / Broca's area BA44 R
4	12.4	56	0	8	Central Opercular Cortex / Secondary somatosensory cortex, Parietal operculum OP4 R
4	12.3	52	-2	8	Central Opercular Cortex / Secondary somatosensory cortex, Parietal operculum OP4 R
4	12.3	40	-14	0	Insular Cortex / Insula Id1 R

3	9.31	-34	48	28	Frontal Pole
2	8.49	0	-24	28	Posterior cingulate gyrus
1	7.75	4	-38	-44	Brainstem
1	6.97	-4	-38	-44	Brainstem

Supplementary table 11. Peak negative brain activation associated with low intensity heat stimuli

Cluster Index	Z	x	y	z	Atlas Location (H/J)
1	11.8	-40	-70	36	Superior lateral occipital cortex / Inferior parietal lobule PGp L
1	11.7	4	-60	20	Precuneous Cortex
1	11.6	-4	-52	28	Posterior cingulate gyrus
1	11.5	-6	-54	20	Precuneous cortex
1	11.4	4	-52	26	Posterior cingulate gyrus
1	11.3	-2	-56	24	Precuneous Cortex

Supplementary table 12. Peak brain activation associated with difference between high and low intensity heat stimuli (high > low)

Cluster Index	Z	x	y	z	Atlas Location (H/J)
6	13.1	54	6	6	Precentral gyrus / Broca's area BA44 R
6	12.7	56	0	8	Central opercular cortex / Secondary somatosensory cortex, Parietal operculum OP4 R
6	12.5	50	-2	6	Central Opercular Cortex / Secondary somatosensory cortex, Parietal operculum OP4 R
6	12.5	40	2	10	Insular Cortex
6	12.5	40	10	4	Central Opercular Cortex
6	12.4	34	6	10	Insular Cortex
5	9.36	-32	44	30	Frontal Pole
4	5.84	56	-62	4	Inferior lateral occipital cortex / Visual cortex V5 R
3	7.63	0	-26	28	Posterior cingulate gyrus
2	5.52	-6	-82	42	Cuneal Cortex / Superior parietal lobule 7P L
1	5.02	14	-78	42	Precuneous Cortex / Superior parietal lobule 7P R

Supplementary table 13. Peak brain activation associated with difference between high and low intensity heat stimuli (low > high)

Cluster Index	Z	x	y	z	Atlas Location (H/J)
3	12.2	6	-58	22	Precuneous Cortex
3	12.2	8	-56	18	Precuneous Cortex
3	12.2	-18	34	48	Superior Frontal Gyrus

3	12.2	10	-56	22	Precuneous Cortex
3	12.1	30	-32	-18	Posterior parahippocampal gyrus / Hippocampus subiculum R
3	12	-34	-72	36	Superior lateral occipital cortex / Inferior parietal lobule PGp L
2	6.74	-46	-28	68	Postcentral gyrus
2	4.94	-54	-16	58	Postcentral gyrus
1	5.8	-68	-4	28	Postcentral gyrus
1	5.22	-64	-6	34	Postcentral gyrus

Supplementary table 14. Peak positive brain activation associated with low intensity cold stimuli

Cluster Index	Z	x	y	z	Atlas Location (H/J)
7	7.26	58	10	18	Precentral Gyrus/ Broca's area BA44 R
7	7.25	60	12	14	Inferior Frontal Gyrus, pars opercularis / Broca's area BA44R
7	7.22	-44	8	-6	Insular Cortex/ Acoustic radiation L
7	7.09	32	22	10	Frontal Operculum Cortex
7	6.93	-34	2	10	Insular Cortex
7	6.88	-30	18	10	Insular Cortex
6	7.64	4	16	54	Superior Frontal Gyrus / Premotor cortex BA6 R
6	7.61	2	16	48	Paracingulate Gyrus/ Premotor cortex BA6 R
6	7.49	4	22	40	Paracingulate Gyrus
6	6.47	-4	20	34	Anterior Cingulate Gyrus
6	6.34	-4	16	36	Anterior Cingulate Gyrus
6	5.15	4	36	42	Superior Frontal Gyrus
5	5.59	-46	38	10	Frontal Pole / Broca's area BA45 L
5	5.51	-38	38	12	Frontal Pole / Broca's area BA45 L
5	5.39	-46	42	10	Frontal Pole / Broca's area BA45 L
5	5.33	-28	50	26	Frontal Pole
5	4.43	-36	50	12	Frontal Pole
5	4.36	-34	52	16	Frontal Pole
4	6.17	-38	-56	-30	Cerebellum
4	5.45	-28	-68	-26	Cerebellum
4	3.71	-48	-62	-32	Cerebellum
4	3.63	-48	-56	-34	Cerebellum
3	4.64	-42	-18	52	Precentral Gyrus / Primary somatosensory cortex BA3b L
3	4.46	-40	-24	54	Postcentral Gyrus / Primary somatosensory cortex BA3b L
3	4.01	-36	-4	62	Middle Frontal Gyrus / Premotor cortex BA6 L
3	4	-36	-24	64	Postcentral Gyrus / Premotor cortex BA6 L
3	3.83	-38	-20	64	Precentral Gyrus / Premotor cortex BA6 L
3	3.82	-40	-6	58	Precentral Gyrus / Premotor cortex BA6 L
2	4.33	-40	-92	-6	Occipital Pole / Visual cortex V4 L
2	4.04	-36	-94	2	Occipital Pole / Visual cortex V3V L
2	3.97	-34	-94	6	Occipital Pole / Visual cortex V3V L
2	3.92	-30	-94	-4	Occipital Pole / Visual cortex V3V L
2	3.9	-30	-98	-6	Occipital Pole / Visual cortex V3V L

2	3.9	-30	-90	6	Inferior Lateral Occipital Cortex / Visual cortex V3V L
1	4.16	18	-102	-2	Occipital Pole / Visual cortex V1 BA17 R
1	4.15	32	-92	-10	Occipital Pole / Visual cortex V3V R
1	3.84	24	-100	-6	Occipital Pole / Visual cortex V1 BA17 R
1	3.81	28	-98	-6	Occipital Pole / Visual cortex V2 BA18 R
1	3.69	20	-98	-8	Occipital Pole / Visual cortex V1 BA17 R
1	3.5	34	-96	-12	Occipital Pole / Visual cortex V2 BA18 R

Supplementary table 15. Peak negative brain activation associated with low intensity cold stimuli

Cluster Index	Z	x	y	z	Atlas Location (H/J)
7	7.39	-14	-60	14	Precuneous Cortex / Visual cortex V2 BA18 L
7	7.16	14	-54	14	Precuneous Cortex / Visual cortex V1 BA17 R
7	7.15	18	-54	20	Precuneous Cortex
7	6.83	-4	-58	16	Precuneous Cortex
7	6.79	8	-62	22	Precuneous Cortex / Superior parietal lobule 7M R
7	6.61	-34	-76	36	Superior Lateral Occipital Cortex / Inferior parietal lobule PGp L
6	6.16	-10	60	6	Frontal Pole
6	6.02	4	64	10	Frontal Pole
6	5.04	-6	54	0	Paracingulate Gyrus
6	4.96	-14	54	0	Paracingulate Gyrus
6	4.78	-16	64	10	Frontal Pole
6	4.36	8	52	0	Paracingulate Gyrus
5	5.39	-22	26	40	Superior Frontal Gyrus
5	4.32	-18	16	44	Superior Frontal Gyrus
5	4.2	-24	14	40	Superior Frontal Gyrus
5	4.11	-28	16	42	Middle Frontal Gyrus
5	3.87	-34	34	48	Middle Frontal Gyrus
5	3.72	-30	18	36	Middle Frontal Gyrus
4	5.48	50	-2	-18	Anterior Superior Temporal Gyrus / Insula Id1 R
4	5.25	58	2	-14	Anterior Superior Temporal Gyrus
4	5.25	54	0	-12	Anterior Superior Temporal Gyrus
4	5.07	50	6	-22	Temporal Pole
4	5.02	44	10	-28	Temporal Pole
4	5.01	46	12	-24	Temporal Pole
3	5.29	20	20	44	Superior Frontal Gyrus
3	4.92	22	30	40	Superior Frontal Gyrus
3	4.18	18	36	46	Frontal Pole / Premotor cortex BA6 R
3	3.9	18	38	36	Frontal Pole
2	5.09	-52	-10	-14	Anterior Middle Temporal Gyrus
2	4.93	-60	-8	-8	Anterior Middle Temporal Gyrus
2	4.88	-56	-4	-12	Anterior Superior Temporal Gyrus
2	4.56	-54	2	-14	Anterior Superior Temporal Gyrus
2	4.09	-56	-4	-20	Anterior Middle Temporal Gyrus
2	3.95	-60	-12	-14	Posterior Middle Temporal Gyrus
1	5.18	8	-50	-44	Cerebellum
1	4.04	-8	-48	-42	Cerebellum

1	4	-6	-54	-42	Cerebellum

Supplementary table 16. Peak brain activation associated with difference between high and low intensity cold stimuli (high > low)

Cluster Index	Z	x	y	z	Atlas Location (H/J)
7	8.35	56	10	4	Inferior Frontal Gyrus, pars opercularis / Broca's area BA44R
7	8.33	-42	14	-2	Insular Cortex
7	8.22	40	-2	-6	Insular Cortex / Inferior occipito-frontal fascicle R
7	8.07	34	20	0	Insular Cortex
7	7.53	-62	-22	24	Postcentral Gyrus / Inferior parietal lobule PFop L
7	7.52	-40	0	-2	Insular Cortex
6	8.11	0	20	44	Paracingulate Gyrus
6	6.79	2	-24	30	Posterior Cingulate Gyrus / Cingulum R
6	5.73	2	28	60	Superior Frontal Gyrus / Premotor cortex BA6 R
6	4.91	2	4	72	Juxtapositional Lobule Cortex / Premotor cortex BA6 R
6	4.24	6	-4	72	Juxtapositional Lobule Cortex / Premotor cortex BA6 R
6	4.17	6	14	70	Superior Frontal Gyrus / Premotor cortex BA6 R
5	6.22	-34	-58	-30	Cerebellum
5	6.06	-24	-66	-30	Cerebellum
5	5.72	-28	-70	-24	Cerebellum
5	5.7	-46	-60	-32	Cerebellum
5	5.44	-10	-76	-28	Cerebellum
5	5.3	-38	-74	-26	Occipital Fusiform Gyrus, Cerebellum
4	4.75	34	-70	-26	Occipital Fusiform Gyrus, Cerebellum
4	4.58	38	-74	-26	Inferior Lateral Occipital Cortex, Cerebellum
4	4.37	38	-60	-28	Cerebellum
4	3.84	38	-56	-30	Cerebellum
4	3.76	48	-66	-30	Cerebellum
4	3.61	48	-60	-32	Cerebellum
3	4.9	-26	-100	-4	Occipital Pole / Visual cortex V3V L
3	3.96	-36	-96	-10	Occipital Pole / Visual cortex V4 L
3	3.95	-32	-94	-12	Occipital Pole / Visual cortex V3V L
3	3.89	-32	-94	0	Occipital Pole / Visual cortex V3V L
3	3.81	-16	-100	-8	Occipital Pole / Visual cortex V2 BA18 L
3	3.79	-34	-96	-4	Occipital Pole / Visual cortex V3V L
2	4.11	-40	-22	64	Precentral Gyrus / Premotor cortex BA6 L
2	3.96	-42	-20	52	Postcentral Gyrus / Primary somatosensory cortex BA3b L
2	3.94	-38	-22	48	Postcentral Gyrus / Primary somatosensory cortex BA3b L
2	3.88	-34	-26	60	Postcentral Gyrus / Corticospinal tract L
2	3.84	-40	-18	64	Precentral Gyrus / Premotor cortex BA6 L
1	4.25	-8	-16	-2	Thalamus
1	3.75	4	-22	-2	Thalamus
1	3.68	0	-26	-2	Thalamus
1	3.56	10	-14	-4	Thalamus
1	3.41	-10	-16	10	Thalamus

Supplementary table 17. Peak brain activation associated with difference between high and low intensity cold stimuli (low > high)

Cluster Index	Z	x	y	z	Atlas Location (H/J)
3	7.31	-38	-78	24	Superior Lateral Occipital Cortex / Inferior parietal lobule PGp L
3	7.19	26	-36	-12	Posterior Parahippocampal Gyrus / Hippocampus subiculum R
3	7.18	-34	-44	-10	Posterior Temporal Fusiform Cortex / Optic radiation L
3	7.16	-34	-78	30	Superior Lateral Occipital Cortex / Inferior parietal lobule PGp L
3	7.14	-36	-66	26	Superior Lateral Occipital Cortex / Inferior parietal lobule PGp L
3	7.09	42	-70	26	Superior Lateral Occipital Cortex / Inferior parietal lobule PGp R
2	5.6	8	-52	-44	Cerebellum
2	4.96	18	-52	-44	Cerebellum
2	4.29	8	-50	-38	Cerebellum
2	4	10	-44	-46	Cerebellum
1	3.98	-2	4	-10	Subcallosal Cortex (ventricle?)
1	3.9	0	20	-2	Subcallosal Cortex / Callosal body
1	3.88	-2	10	-12	Subcallosal Cortex
1	3.71	0	14	-6	Subcallosal Cortex
1	3.68	-2	16	-10	Subcallosal Cortex
1	3.53	2	26	2	Subcallosal Cortex / Callosal body

Supplementary table 18. Peak positive brain activation associated with low intensity auditory stimuli

Cluster Index	Z	x	y	z	Atlas Location (H/J)
9	9.89	50	-8	2	Heschl's Gyrus / Primary auditory cortex TE1.0 R
9	9.58	50	-22	8	Heschl's Gyrus / Primary auditory cortex TE1.1 R
9	9.56	38	18	0	Insular Cortex / Inferior occipito-frontal fascicle R
9	9.54	50	8	4	Precentral Gyrus / Broca's area BA44 R
9	9.54	42	20	0	Frontal Operculum Cortex / Right Cerebral Cortex
9	8.95	62	-24	12	Planum Temporale / Inferior parietal lobule PF R
8	9.94	-40	-30	10	Planum Temporale / Primary auditory cortex TE1.1 L
8	9.5	-48	-26	8	Heschl's Gyrus / Primary auditory cortex TE1.0 L
8	9.42	-46	-20	6	Heschl's Gyrus / Primary auditory cortex TE1.0 L
8	9.02	-48	-14	2	Heschl's Gyrus / Primary auditory cortex TE1.0 L
8	8.57	-38	18	-2	Insular Cortex
8	8.54	-54	-36	16	Planum Temporale / Inferior parietal lobule PFcm L
7	8.72	4	16	52	Paracingulate Gyrus, Superior Frontal Gyrus / Premotor cortex BA6 R
7	8.49	4	8	58	Juxtapositional Lobule Cortex / Premotor cortex BA6 R
7	5.96	14	8	68	Superior Frontal Gyrus / Premotor cortex BA6 R

7	5.28	8	28	30	Paracingulate Gyrus
7	3.52	-8	22	32	Paracingulate Gyrus
6	6.17	-32	50	30	Frontal Pole
6	6.13	-28	46	20	Frontal Pole
6	6.08	-34	42	26	Frontal Pole
6	5.87	-36	46	24	Frontal Pole
6	5.87	-44	40	-2	Frontal Pole / Broca's area BA45 L
6	5.29	-38	36	30	Middle Frontal Gyrus
5	8.06	-30	-70	-24	Cerebellum
5	6.98	-30	-60	-30	Cerebellum
5	6.53	-46	-58	-32	Cerebellum
5	5.91	-8	-78	-20	Occipital Fusiform Gyrus, Cerebellum
5	5.67	-8	-78	-28	Cerebellum
4	5.47	-34	-26	66	Postcentral gyrus, Precentral gyrus / Premotor cortex BA6L
4	4.81	-42	-2	44	Precentral Gyrus / Premotor cortex BA6 L
4	4.56	-38	-24	54	Postcentral gyrus, Precentral gyrus/ Primary somatosensory cortex BA3bL, Primary motor cortex BA4aL
4	4.47	-42	-18	58	Precentral Gyrus / Premotor cortex BA6 L, Primary motor cortex BA4a L
4	4.09	-50	6	38	Precentral Gyrus / Premotor cortex BA6 L, Broca's area BA44 L
4	3.92	-44	4	46	Middle Frontal Gyrus / Premotor cortex BA6 L
3	6.75	2	-22	26	Posterior Cingulate Gyrus
3	6.48	2	-30	24	Posterior Cingulate Gyrus
2	4.59	-24	-4	4	Putamen
2	4.49	-24	8	0	Putamen
2	3.42	-18	18	-2	Nucleus Caudate
1	4.45	14	-76	42	Precuneous Cortex / Superior parietal lobule 7P R
1	4	14	-76	54	Superior Lateral Occipital Cortex / Superior parietal lobule 7P R
1	3.99	12	-80	52	Superior Lateral Occipital Cortex / Superior parietal lobule 7P R
1	3.8	18	-74	60	Superior Lateral Occipital Cortex / Superior parietal lobule 7P R

Supplementary table 19. Peak negative brain activation associated with low intensity auditory stimuli

Cluster Index	Z	x	y	z	Atlas Location (H/J)
1	9.61	-14	-60	22	Precuneous Cortex / Visual cortex V2 BA18 L
1	9.37	4	-60	24	Precuneous Cortex
1	8.93	-2	-54	32	Posterior Cingulate Gyrus / Superior parietal lobule 7A L
1	8.79	-4	-56	26	Precuneous Cortex
1	8.74	12	-60	24	Precuneous Cortex
1	8.27	-36	-80	30	Superior Lateral Occipital Cortex / Inferior parietal lobule PGp L

Supplementary table 20. Peak brain activation associated with difference between high and low intensity auditory stimuli (high > low)

Cluster Index	Z	x	y	z	Atlas Location (H/J)
7	9.75	50	-6	2	Heschl's Gyrus / Primary auditory cortex TE1.0 R
7	9.62	56	-18	8	Planum Temporale / Primary auditory cortex TE1.0 R
7	9.62	60	-22	12	Planum Temporale/ Secondary somatosensory cortex, Parietal operculum OP1 R
7	9.59	50	-22	10	Heschl's Gyrus / Primary auditory cortex TE1.1 R
7	8.58	52	8	2	Central Opercular Cortex, Precentral Gyrus / Broca's area BA44 R
7	8.56	44	20	-2	Frontal Operculum Cortex / Broca's area BA45 R
6	9.95	-40	-30	12	Planum Temporale / Primary auditory cortex TE1.1 L
6	9.77	-48	-26	6	Planum Temporale, Heschl's Gyrus / Primary auditory cortex TE1.0 L
6	8.37	-50	-8	2	Planum Polare / Secondary somatosensory cortex, Parietal operculum OP4 L
6	7.81	-42	-16	-6	Planum Polare / Insula Id1 L
6	7.55	-48	16	-6	Frontal Operculum Cortex / Broca's area BA45 L
6	7.51	-40	-4	-14	Insular Cortex/ GM Insula Id1 L
5	8.22	2	20	46	Paracingulate Gyrus / Premotor cortex BA6 R
5	7.06	4	6	60	Juxtapositional Lobule Cortex / Premotor cortex BA6 R
5	6.81	4	32	46	Superior Frontal Gyrus / Premotor cortex BA6 R
5	5.99	4	4	72	Juxtapositional Lobule Cortex / Premotor cortex BA6 R
5	4.28	16	10	66	Superior Frontal Gyrus / Premotor cortex BA6 R
5	4.26	14	6	70	Superior Frontal Gyrus / Premotor cortex BA6 R
4	6.95	-32	-66	-26	Cerebellum
4	5.97	-12	-74	-30	Cerebellum
4	5.67	-10	-78	-22	Occipital Fusiform Gyrus
4	5.05	-42	-70	-26	Occipital Fusiform Gyrus
4	4.18	-38	-80	-20	Inferior Lateral Occipital Cortex / Visual cortex V4 L
4	3.98	-46	-60	-28	Temporal Occipital Fusiform Cortex
3	5.09	-10	6	8	Nucleus Caudate
3	4.96	-8	0	12	Nucleus Caudate
3	4.78	8	2	8	Nucleus Caudate
3	4.73	-22	2	-12	Amygdala
3	4.68	12	2	14	Nucleus Caudate
3	4.59	16	14	2	Nucleus Caudate
2	7.06	-2	-26	28	Posterior Cingulate Gyrus
1	4.45	36	-72	-22	Occipital Fusiform Gyrus
1	4.38	34	-70	-26	Occipital Fusiform Gyrus
1	3.99	34	-60	-32	Cerebellum
1	3.75	36	-52	-32	Cerebellum

Supplementary table 21. Peak brain activation associated with difference between high and low intensity auditory stimuli (low > high)

Cluster Index	Z	x	y	z	Atlas Location (H/J)
1	7.89	-32	-40	-8	Posterior Parahippocampal Gyrus / Optic radiation L
1	7.88	8	-62	24	Precuneous Cortex / Superior parietal lobule 7M R
1	7.83	-6	-62	20	Precuneous Cortex / Superior parietal lobule 7M L
1	7.82	-10	-58	20	Precuneous Cortex
1	7.56	-44	-74	34	Superior Lateral Occipital Cortex / Inferior parietal lobule PGp L
1	7.28	-36	-78	30	Superior Lateral Occipital Cortex / Inferior parietal lobule PGp L

Supplementary Analyses

Supplementary Analysis 1: Relationship between brain activation in response to high intensity heat stimuli and individual pain intensity rating within pain sensitivity classes

Methods: The main GLM analysis, i.e. investigating the relationship between brain activation in response to high intensity heat stimuli and individual pain intensity rating, was repeated within pain sensitivity classes to investigate if the relationship between pain intensity rating and brain activation associated with high intensity heat stimuli could be established within classes of pain sensitivity.

Results: Results of these analyses did not show any relationship between brain activations associated with high intensity heat stimulation and pain intensity ratings.

Interpretation & implications: These results support our main results, i.e. interindividual variability in pain sensitivity is not associated with brain response to high intensity heat stimuli.

Supplementary Analysis 2: Effect of covariates of no interest on the relationship between brain activation associated with high intensity heat stimuli and individual pain intensity rating

Methods: the main GLM analysis, i.e. investigating the relationship between brain activation in response to high intensity heat stimuli and individual pain intensity rating, was repeated while including all the acquired psychological and demographics data, as well as the head motion parameters (RMS and FWD) as covariates of no interest.

Results: Results of this analysis did not show any relationship between interindividual pain intensity and brain activation associated with high intensity heat stimuli.

Interpretation & implications: These results further support our main findings that inter-individual pain sensation is dissociated from brain activation associated with high intensity heat stimuli.

Supplementary Analysis 3: Relationship between brain activation in response to high intensity heat stimuli and individual pain intensity rating in the adult subsample of our cohort

Methods: the main GLM analysis, i.e. investigating the relationship between brain activation in response to high intensity heat stimuli and individual pain intensity rating, was repeated without the adolescent participants to confirm that their inclusion did not affect the main findings.

Results: Results of this analysis did not yield any significant relationship between brain activation associated with high intensity heat stimuli and individual pain intensity ratings.

Interpretation & implications: These results suggest that the inclusion of adolescent participants did not affect our main findings.

Supplementary Analysis 4: Relationship between brain activation in response to high intensity heat stimuli and individual pain intensity rating in a subsample matching the one of Coghill et al. (2003)

Methods: the main GLM analysis, i.e. investigating the relationship between brain activation in response to high intensity heat stimuli and individual pain intensity rating, was repeated in a subsample matching the one from the original study (Coghill et al., 2003). The samples were matched by sex, race, pain sensitivity, and averaged age.

Results: Results of this analysis were consistent with that of the whole sample. Robust effects of noxious heat stimulation (mean effect) were detected in a similar set of regions to that of that of the whole sample. The analysis of the subset did not reveal any relevant relationship between brain activation associated with high intensity heat stimuli and individual pain intensity ratings, although an anomalous cluster was identified in the supramarginal gyrus. Given the number of tests that we have run in both the main and supplementary analyses, in combination with the location, we feel that this is likely a false positive.

Interpretation & implications: These results support our main findings and suggest that the results reported in Coghill et al. (2003) were likely due to an error inherent to the small sample size.

Supplementary analysis 5: Test-retest reliability

Methods: A Bland-Altman analysis was performed to assess test-retest reliability of our pain intensity ratings between the two sessions.

Results: This analysis reveals a difference of approximately 1.5 VAS unit between the two sessions, with pain intensity being typically rated as greater in the first session than in the second session.

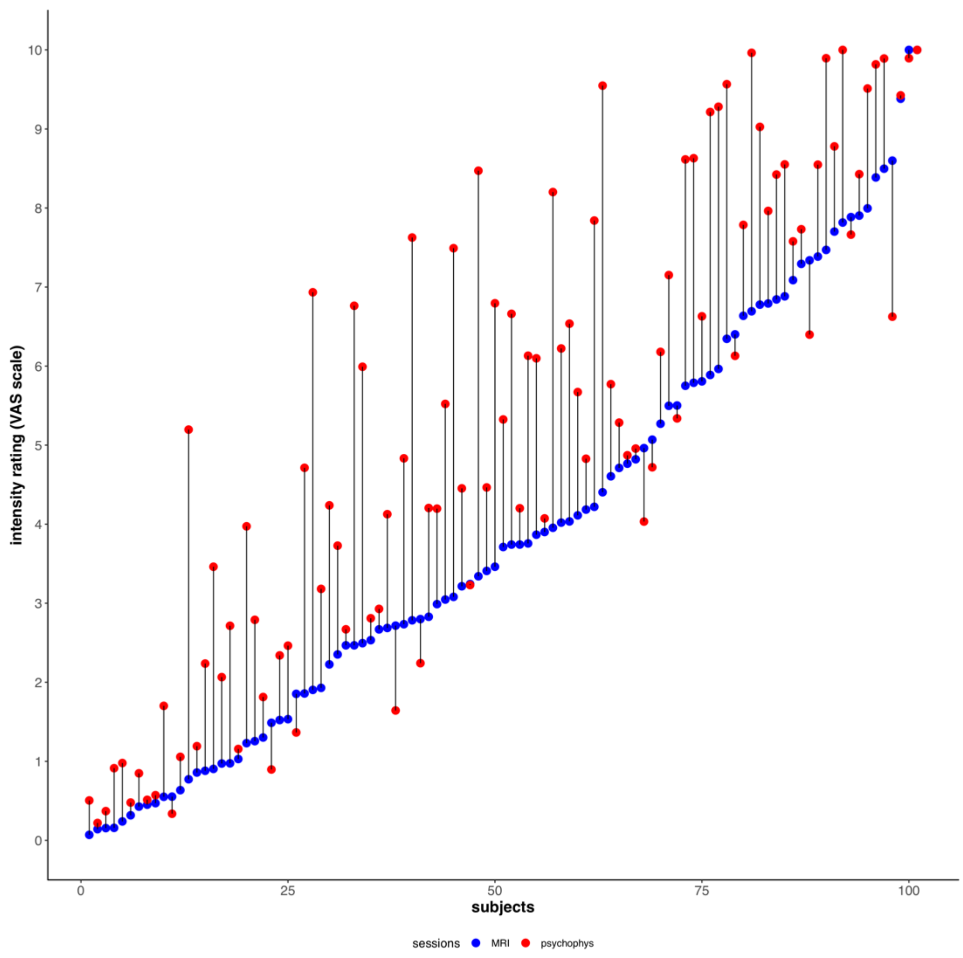

Interpretation & implications: These results show variation over time in pain sensation with sensations being rated as greater in the first session. This systematic bias could be due to an exposure effect or to the MRI environment, in which the second session took place. However, this bias is unlikely to have affected our findings or our conclusions, given that our main analyses were performed on data and ratings acquired within the same session.

Supplementary analysis 6: inter-individual correlations between pain intensity and pain unpleasantness ratings and psychological variables.

Methods: Pearson's correlations were performed to ensure that our results were not impacted by our approach including a mixture model analysis. Correlations were performed between individual ratings of pain intensity and unpleasantness and psychological variables.

Results: No significant correlation was found between pain ratings and psychological variable, as shown in the table below.

	**pain intensity**		**pain unpleasantness**	
**covariate**	**r**	**p**	**r**	**p**
sleepiness	0.194102	0.05178	0.176185	0.078
impulsiveness	0.153968	0.1242	0.085223	0.3968
experience of discrimination	0.041456	0.6806	0.003987	0.9684
functional disability	0.136783	0.6716	0.166837	0.6043
mindfulness	-0.08802	0.459	-0.047263	0.6913
sleep quality	-0.01362	0.9089	-0.080737	0.4971
screen for child anxiety	0.202959	0.527	0.065364	0.8401
positive affect	-0.17682	0.07691	-0.145226	0.1473
negative affect	0.085882	0.3931	0.099718	0.3211
PROMIS anxiety	0.060226	0.6128	0.080758	0.497
PROMIS depression	-0.06856	0.5644	-0.078965	0.5066
catastrophizing	0.090861	0.4446	0.004623	0.969
PROMIS pain interference	0.193011	0.1018	0.1894	0.1085

Interpretation: Results of these correlations further support our results presented in the main manuscript.

Supplementary analysis 7: differences in pain intensity and pain unpleasantness ratings within demographic variables.

Methods: additional tests were performed to ensure that our results on differences in demographic variables between classes of pain sensitivity were not impacted by our approach including a mixture model analysis. A two-sided independent sample t-test was performed to compare differences in pain intensity and unpleasantness ratings between sex. In addition, two one-way ANOVAs were performed to compare differences in pain intensity and unpleasantness ratings between races. Finally, a Pearson correlation was performed between individual ratings of pain intensity and unpleasantness and age.

Results: Results of the t-test did not show any significant difference in pain ratings between sex (intensity: $t(70.4) = 0.21$, $p = 0.84$; unpleasantness: $t(70.5) = 0.17$, $p = 0.86$). Results of the ANOVA did not show any differences in pain ratings between races (intensity: $F(93) = 0.309$, $p = 0.579$; unpleasantness: $F(93) = 0.426$, $p = 0.516$). Finally, results of the correlation did not reveal any relationship between pain ratings and age (intensity: $r(99) = 0.16$, $p = 0.1$; unpleasantness: $r(99) = 0.13$, $p = 0.2$).

Interpretation: These results further support the results presented in the main manuscript.

Supplementary analysis 8: inter-individual correlations between pain intensity and pain unpleasantness ratings and discrimination thresholds.

Methods: Pearson's correlations were performed to ensure that our results were not impacted by our approach including a mixture model analysis. Correlations were performed between individual ratings of pain intensity and 43-ascending and 49-descending discrimination thresholds for intensity and between individual ratings of pain unpleasantness and 43-

ascending and 49-descending discrimination thresholds for pain unpleasantness. Bonferroni correction was applied when appropriate.

Results: Before Bonferroni correction, significant correlations were found between pain intensity ratings and 43-ascending discrimination threshold for intensity (r(99) = -0.26, p = 0.007). Similarly, significant correlations were found between pain unpleasantness and 43-ascending discrimination threshold for unpleasantness (r(99) = -0.39, p = 0.00006), and between pain unpleasantness and 49-descending discrimination threshold for unpleasantness (r(99) = -0.24, p = 0.015).

After Bonferroni correction, the correlation between pain intensity ratings and 43-ascending discrimination threshold for intensity remained significant. Similarly, the correlation between pain unpleasantness ratings and 43-ascending discrimination threshold for unpleasantness remained significant after correlation.

Correlations' results are displayed in the two tables below. Correlations that remained significant after correction are indicated by *.

Interpretation: Results of these correlation further support our results presented in the main manuscript, suggesting that the ability to discriminate the perception of noxious stimuli is associated with individual pain sensitivity.

**pain intensity rating**		
**Covariates**	**r**	**p**
43-ascending discrimination threshold for intensity	-0.26475	0.007463*
49-descending discrimination threshold for intensity	-0.12663	0.207

**pain unpleasantness rating**		
**Covariates**	**r**	**p**

43-ascending discrimination threshold for unpleasantness	-0.38764	0.00006201*
49-descending discrimination threshold for unpleasantness	-0.2418	0.01485

Supplementary analysis 9: inter-individual correlations between pain intensity and pain unpleasantness ratings and head motion parameters in the scanner.

Methods: Pearson's correlations were performed to ensure that our results were not impacted by our approach including a mixture model analysis. Correlations were performed between individual ratings of pain intensity and unpleasantness and head motion parameters, including FWD and RMS.

Results: No significant correlation was found between pain ratings and head motion parameters, as shown in the table below.

**pain intensity**		
**covariates**	**r**	**p**
RMS	0.046637	0.6433
FWD	0.031121	0.7574

**pain unpleasantness**		
**covariates**	**r**	**p**
RMS	0.1478	0.1402
FWD	0.1292	0.1978

Interpretation: Results of these correlations further support our results presented in the main manuscript.

Supplementary discussion

Deactivation in the white matter:

Results from the analyses performed on our fMRI data show task-related decreased activation in white matter. Task-related changes in white matter activation are not very well known, nor studied. It has been previously shown that changes in white matter activation can be associated with tasks, such as visual tasks [1,2] or mindfulness [3,4]. Although this is the first report of decreased white matter activation associated with pain, prior findings of decreased cerebral blood flow associated with painful stimulation have been reported [5] and are in line with our findings. Further studies are needed to confirm and deepen our understanding of this relationship.

1. Gore, J. C. *et al.* Functional MRI and resting state connectivity in white matter - a mini-review. *Magn Reson Imaging* 63, 1–11 (2019).

2. Gawryluk, J. R., Mazerolle, E. L. & D'Arcy, R. C. N. Does functional MRI detect activation in white matter? A review of emerging evidence, issues, and future directions. *Frontiers in Neuroscience* 8, 145 (2014).

3. Zeidan, F., Baumgartner, J. N. & Coghill, R. C. The neural mechanisms of mindfulness-based pain relief: a functional magnetic resonance imaging-based review and primer. *Pain Reports* 4, e759 (2019).

4. Zeidan, F. *et al.* Mindfulness Meditation-Based Pain Relief Employs Different Neural Mechanisms Than Placebo and Sham Mindfulness Meditation-Induced Analgesia. *Journal of Neuroscience* 35, 15307–15325 (2015).

5. Coghill, R. C., Sang, C. N., Berman, K. F., Bennett, G. J. & Iadarola, M. J. Global cerebral blood flow decreases during pain. *Journal of Cerebral Blood Flow and Metabolism* 18, 141–147 (1998).
