# Supplementary material for: Dissociation between individual differences in self-reported pain intensity and underlying fMRI brain activation
Source: Nat Commun. 2022 Jun 22;13:3569. doi: 10.1038/s41467-022-31039-3 (PMC9218124; doi:10.1038/s41467-022-31039-3)
Supplement: Supplementary file 3 — Reporting Summary [file 41467_2022_31039_MOESM3_ESM.pdf]

nature research

Corresponding author(s): Marie-Eve Hoeppli
Robert C. Coghill

Last updated by author(s): Mar 22, 2022

Reporting Summary

Nature Research wishes to improve the reproducibility of the work that we publish. This form provides structure for consistency and transparency in reporting. For further information on Nature Research policies, see our Editorial Policies and the Editorial Policy Checklist.

Statistics

For all statistical analyses, confirm that the following items are present in the figure legend, table legend, main text, or Methods section.

n/a	Confirmed	
☐	☒	The exact sample size (n) for each experimental group/condition, given as a discrete number and unit of measurement
☐	☒	A statement on whether measurements were taken from distinct samples or whether the same sample was measured repeatedly
☐	☒	The statistical test(s) used AND whether they are one- or two-sided<br>*Only common tests should be described solely by name; describe more complex techniques in the Methods section.*
☐	☒	A description of all covariates tested
☐	☒	A description of any assumptions or corrections, such as tests of normality and adjustment for multiple comparisons
☐	☒	A full description of the statistical parameters including central tendency (e.g. means) or other basic estimates (e.g. regression coefficient) AND variation (e.g. standard deviation) or associated estimates of uncertainty (e.g. confidence intervals)
☐	☒	For null hypothesis testing, the test statistic (e.g. F, t, r) with confidence intervals, effect sizes, degrees of freedom and P value noted<br>*Give P values as exact values whenever suitable.*
☒	☐	For Bayesian analysis, information on the choice of priors and Markov chain Monte Carlo settings
☐	☒	For hierarchical and complex designs, identification of the appropriate level for tests and full reporting of outcomes
☐	☒	Estimates of effect sizes (e.g. Cohen's d, Pearson's r), indicating how they were calculated

Our web collection on statistics for biologists contains articles on many of the points above.

Software and code

Policy information about availability of computer code

Data collection	A custom IDL software was used to collect ratings during the MRI session.<br>A Medoc pathway was used to produce heat and cold stimulus.<br>Auditory stimulus were delivered using iTunes.<br>Brain images were acquired using a 3T Philips scanner and a 32-channel head coil.<br>REDCap was used to collect psychological and demographic data, as well as some psychophysical data
Data analysis	Analyses of the psychophysical data were done using Rstudio version 3.6.2 and SPSS version 25.<br>FSL version 6.0 and associated tools were used for all preprocessing of the fMRI data and for the univariate analyses. Matlab 2016a and SPM 12 were used for the multivariate analyses.

For manuscripts utilizing custom algorithms or software that are central to the research but not yet described in published literature, software must be made available to editors and reviewers. We strongly encourage code deposition in a community repository (e.g. GitHub). See the Nature Research guidelines for submitting code & software for further information.

Data

Policy information about availability of data

All manuscripts must include a data availability statement. This statement should provide the following information, where applicable:

- Accession codes, unique identifiers, or web links for publicly available datasets
- A list of figures that have associated raw data
- A description of any restrictions on data availability

Data is available upon request. Source data are provided with this paper and activation maps have been uploaded to Github (https://github.com/coghill-painlab/IDP_fMRI_activationMaps).

Field-specific reporting

Please select the one below that is the best fit for your research. If you are not sure, read the appropriate sections before making your selection.

[x] Life sciences [ ] Behavioural & social sciences [ ] Ecological, evolutionary & environmental sciences

For a reference copy of the document with all sections, see nature.com/documents/nr-reporting-summary-flat.pdf

Life sciences study design

All studies must disclose on these points even when the disclosure is negative.

Sample size	Sample size calculation for fMRI studies are extremely challenging, and no sample size calculations were performed for this aim of the grant application. Our initial study was published with n=12. Individual differences in pain sensitivity are highly robust, and our current n is 101. Adequate power is further confirmed by the presence of robust responses to graded stimulus intensities across noxious heat, noxious cold, and auditory stimuli.
Data exclusions	8 participants were excluded from all analyses because of insufficient quality of the fMRI images or incidental findings of abnormalities on MRI.<br>Due to technical issues during the MRI session, 28 additional participants had missing data in the cold series and 4 had missing data in the auditory series. These additional participants were selectively excluded from the cold, respectively auditory, analyses.
Replication	We acquired and analyzed a single separate cohort of participants (n=34) not included in the present analysis. Findings of individual differences are identical.
Randomization	All participants received the same stimuli. However, MRI sequences were pseudo-randomized and all stimuli were delivered in a pseudo-randomized order.
Blinding	This is a single-group study. Participants were blinded to stimulus intensity. Blinding was not possible during stimulus delivery and data acquisition. Moreover, all analyses were accomplished by an individual separate from the individuals collecting the data. Therefore, the researcher performing the analyses was blinded to participants' pain expression and behaviors. However, given the nature of the analyses, blinding of that researcher to participants' pain sensitivity was not possible.

Reporting for specific materials, systems and methods

We require information from authors about some types of materials, experimental systems and methods used in many studies. Here, indicate whether each material, system or method listed is relevant to your study. If you are not sure if a list item applies to your research, read the appropriate section before selecting a response.

Materials & experimental systems

n/a	Involved in the study	
[x]	[ ]	Antibodies
[x]	[ ]	Eukaryotic cell lines
[x]	[ ]	Palaeontology and archaeology
[x]	[ ]	Animals and other organisms
[ ]	[x]	Human research participants
[x]	[ ]	Clinical data
[x]	[ ]	Dual use research of concern

Methods

n/a	Involved in the study	
[x]	[ ]	ChIP-seq
[x]	[ ]	Flow cytometry
[ ]	[x]	MRI-based neuroimaging

Human research participants

Policy information about studies involving human research participants

Population characteristics	The age of the 143 healthy individuals included in this study was ranged from 14 to 44 years old. The mean (SD) age of the 34 participants included in the FIX training was 28 (6.1). The mean (SD) age of the 101 participants included in the analyses described in this manuscript was 28.5 (7.7). This group of participants included 43 males and 58 females. Exclusion criteria included active neurological or psychiatric disorder that impacted the participant's ability to perform the tasks requested, the presence or history of chronic pain, medications that could interfere with QST or brain function, positive screen for recreational drugs, any serious pathology, substantial uncorrected visual deficit, and any MRI contraindication, such as any metallic implant or braces.
Recruitment	Participants were recruited through flyers and emails disseminated through the community and Cincinnati Children's Hospital and through word to mouth.
Ethics oversight	Participants and parents/legal guardians of minor participants gave their written informed consent and minors provided written assent in accordance with the institutional review board of Cincinnati Children's Hospital Medical Center, which approved the study.

Note that full information on the approval of the study protocol must also be provided in the manuscript.

Magnetic resonance imaging

Experimental design

Design type	task fMRI, block-design
Design specifications	Heat pain: 17 48˚C stimuli and 4 47˚C stimuli.<br>Cold pain: 4 0.5˚C stimuli and one 3˚C stimuli.<br>Auditory task: 5 90dB stimuli and 2 80dB stimuli .<br>All stimuli lasted 10 seconds and were followed by 16-second rating periods and 22-second resting periods.
Behavioral performance measures	Ratings of pain intensity and pain unpleasantness were recorded using a computerized VAS.

Acquisition

Imaging type(s)	functional, structural
Field strength	3T
Sequence & imaging parameters	gradient echo, EPI, FOV: 240x240x136mm, acquisition matrix: 80x78, voxel size: 3x3x4mm, slice orientation: transverse, slice order: ascending, TR/TE/flip angle: 2sec/35msed/90deg
Area of acquisition	whole brain

Diffusion MRI ☐ Used ☒ Not used

Preprocessing

Preprocessing software	the software FSL version 6.0 was used for all preprocessing.
Normalization	Data normalization was performed linearly and non-linearly to high-resolution individual images and a standard template using the FSL tools FLIRT and FNIRT
Normalization template	The standard template MNI152 was used to normalize the data.
Noise and artifact removal	The following denoising steps were applied to the data: motion correction, slice timing correction, high-pass filtering, spatial smoothing, removal of independent components identified as noise by a custom-trained FIX classifier, intensity normalization.
Volume censoring	no volume censoring was applied.

Statistical modeling & inference

Model type and settings	Mass univariate and multivariate analyses (group level only) were performed. Temporal derivatives and filtering were used at the first level. Fixed effects were used at the second individual level. Mixed effects were used at the group 3rd level.
Effect(s) tested	The main effect of high and low intensity stimulation for heat, cold, and auditory paradigms were analyzed. In addition, the association between ratings of pain intensity and activation associated with high intensity stimulus was analyzed at a group level. Finally, the difference in brain activation between high and low intensity stimuli was analyzed using t-tests.

Specify type of analysis: ☒ Whole brain ☐ ROI-based ☐ Both

Statistic type for inference (See Eklund et al. 2016)	Cluster-wise analysis, z>3.1 p<0.05
Correction	Gaussian Random Field Theory

Models & analysis

n/a	Involved in the study
☒	☐ Functional and/or effective connectivity
☒	☐ Graph analysis
☐	☒ Multivariate modeling or predictive analysis

Multivariate modeling and predictive analysis	LASSO-PCR model using brain activation in response to noxious heat stimuli to predict perceived pain intensity ratings. 5-fold test, repeated five times, Bootstrap test with 5000 samples.
